# Oral HIV-Associated Kaposi Sarcoma: A Clinical Study from the Ga-Rankuwa Area, South Africa

**DOI:** 10.1155/2012/873171

**Published:** 2012-09-12

**Authors:** Razia A. G. Khammissa, Liron Pantanowitz, Liviu Feller

**Affiliations:** ^1^Department of Periodontology and Oral Medicine, University of Limpopo, Medunsa Campus, Pretoria 0204, South Africa; ^2^Department of Pathology, University of Pittsburgh Medical Centre, Pittsburgh, PA 15260, USA

## Abstract

*Background*. Kaposi sarcoma (KS) is one of the most common neoplasms diagnosed in HIV-seropositive subjects. Oral involvement is frequent and is associated with a poor prognosis. The aim of this study was to characterize the features of oral HIV-KS in patients from Ga-Rankuwa, South Africa. *Methods*. All cases with confirmed oral HIV-KS treated at the oral medicine clinic in Ga-Rankuwa from 2004 to 2010 were included in this retrospective study. Differences between males and females with oral HIV-KS in relation to HIV infection status, to oral KS presentation and to survival rates were statistically analysed. *Results*. Twenty (54%) of the 37 patients in the study were females and 17 (46%) were males. In 21 patients (57%), the initial presentation of HIV-KS was in the mouth. Other than the fact that females presented with larger (≥10 mm) oral KS lesions (*P* = 0.0004), there were no statistically significant gender differences. Significantly more patients presented with multiple oral HIV-KS lesions than with single lesions (*P* = 0.0003). Nine patients (24%) developed concomitant facial lymphoedema, and these patients had a significantly lower CD4+ T-cell count (28 cells/mm^3^) compared to the rest of the group (130 cells/mm^3^) (*P* = 0.01). The average CD4+ T-cell count of the patients who died (64 cells/mm^3^) was significantly lower (*P* = 0.0004), there were no statistically significant gender differences. Significantly more patients presented with multiple oral HIV-KS lesions than with single lesions (*P* = 0.016) at the time of oral-KS presentation than of those who survived (166  cells/mm^3^). Conclusions: In Ga-Rankuwa, South Africa where HIV-KS is prevalent, oral KS affects similarly males and females. A low CD4+ T-cell count at the time of oral HIV-KS diagnosis and the development of facial lymphoedema during the course of HIV-KS disease portends a poor prognosis.

## 1. Introduction

Kaposi sarcoma (KS) is a multicentric angioproliferative disorder of endothelial origin [1, 2]. KS predominantly affects mucocutaneous sites, but may also affect visceral organs. KS is characterized microscopically by angiogenesis, the presence of spindle-shaped tumour cells, an inflammatory cell infiltrate dominated by mononuclear cells, extravasated erythrocytes, and oedema [3, 4].

There are four clinicoepidemiological variants of KS: classic KS, endemic KS, iatrogenic KS, and HIV-associated KS (HIV-KS). These variants develop in distinct populations of subjects, and in all of them, the mouth may be affected. Human herpes virus 8 (HHV8) is a critical factor, although not on its own sufficient for the development of KS. Other cofactors including profound immune impairment, angiogenic mediators, or genetic predisposition appear to be necessary for the development of KS [5]_._

HIV-KS may develop at any stage of HIV infection including the stage of early HIV-seropositivity, but it is more prevalent at a lower CD4+ T-cell count [6]. It may be mild or life threatening. Aggressive HIV-KS is associated with disseminated lesions, with intraoral exophytic lesions, with facial lymphoedema, and with an increased HHV8 viral load [7, 8]. HIV-KS may sometimes present as an immune reconstitution inflammatory syndrome (IRIS) shortly after the introduction of antiretroviral therapy, in parallel with the improvement of the host immune status [9–11].

 It is estimated that in 22% of HIV-seropositive subjects with KS the initial presentation of HIV-KS is in the mouth, and that in up to 71% of subjects with HIV-KS, sooner or later the mouth will be affected [12, 13]. Oral HIV-KS lesions may be single or multifocal initially present as macules that progress to papulonodular lesions and ultimately become confluent forming large exophytic masses [14]. The mortality rate of patients with oral HIV-KS is greater than patients with only cutaneous HIV-KS. The former has a 24-month median survival rate compared to the latter that has a 72-month median survival rate [15].

HIV-KS is common in African countries where HHV8 infection is endemic, where HIV infection has reached epidemic proportions, and where antiretroviral medication is not always available [16, 17]. In South Africa, the prevalence of HIV infection is estimated to be about 30% [16, 18] and the prevalence of HHV8 infection may reach 40% in some areas [16, 17]. 

Worldwide, HIV-KS affects males more commonly than females [19], but in sub-Saharan Africa where HIV infection is more prevalent among young females than among young males, the frequency of HIV-KS disease among females has accordingly increased rapidly [4, 19, 20]. However, the female-to-male ratio, age distribution, and the course of oral HIV-KS in South Africa are not well defined. 

The aim of this retrospective study was to characterise the clinical features and course of oral HIV-KS in patients attending the oral medicine clinic at the School of Oral Health Sciences, University of Limpopo, Medunsa campus, South Africa, and to investigate differences between females and males with oral HIV-KS with regard to their CD4+ T-cell count, to the clinical presentation of oral HIV-KS and to survival rate.

## 2. Materials and Methods

Approval of the study was obtained from the Medical Research and Ethics Committee of the University of Limpopo, Medunsa campus, Pretoria, South Africa (MREC 0/212/2010 : PG). All the files of patients with histologically and clinically confirmed oral HIV-KS treated in the Department of Periodontology and Oral Medicine, School of Oral Health Sciences, University of Limpopo, Medunsa campus, from January 2004 until November 2010 were retrieved. 

In this retrospective study, the diagnosis of KS was confirmed by microscopic examination of incisional biopsy specimens by an oral pathologist; the HIV-serostatus of the patients was determined by enzyme-linked immunosorbent assay (ELISA) and Western blot. 

Data were recorded with regard to patient age, race, and gender; the oral site affected by HIV-KS; the clinical appearance of oral HIV-KS lesions; the period of HIV-seropositivity before a KS diagnosis was rendered; the CD4+ T-cell count at the time of HIV diagnosis and when HIV-KS was diagnosed; whether patients received highly active antiretroviral therapy (HAART) at the time of oral HIV-KS diagnosis, or thereafter; any KS involvement on the skin; the presence of facial lymphoedema; the presence of an immune reconstitution inflammatory syndrome (IRIS); the treatment modality used for oral HIV-KS; the course and response to treatment of oral HIV-KS disease; the survival period of patients from the time oral HIV-KS was diagnosed until the end of the observation period; for those who died during the observation period, the time that had elapsed from oral HIV-KS diagnosis to death. 

IRIS-associated oral HIV-KS was diagnosed when there was worsening of pre-existing oral HIV-KS, or when there was development of new oral HIV-KS lesions, shortly after the introduction of HAART in parallel with an improvement in the immune status.

The presence of any pertinent medical information or HIV-associated oral diseases other than oral HIV-KS was also documented. 

The clinical appearance of oral HIV-KS was categorised into macular, papular, nodular, and exophytic lesions. The lesions were classified into three size groups: smaller than 10 mm; between 10 mm and 50 mm and larger than 50 mm. The lesions of oral HIV-KS were categorised as solitary or multifocal. The oral site affected and the number of lesions per site was documented. Lesions affecting the upper and lower retromolar area and the soft palate were categorised as oropharyngeal lesions. 

HAART comprised nevirapine, lamivudine, and stavudine. Local cytotoxic chemotherapy consisted of intralesional bleomycin. Systemic cytotoxic chemotherapy comprised combination of low-dose intravenous vincristine, bleomycin, and daunorubicin. 

### 2.1. Statistical Analysis

Differences between proportion were statistically tested using the Chi-squared test, two-sided *P* < 0.05.

## 3. Results

The study population comprised 37 patients diagnosed with oral HIV-KS, all of whom were black persons. The mean age at the time of oral HIV-KS diagnosis was 33.4 years (Table 1). Two patients were children aged 10 and 11 years. Seventeen males (46%) and 20 females (54%) were affected (M : F = 1 : 1.2). Nine patients had a history of smoking tobacco (Table 1). 

In 21 patients (57%) the initial presentation of HIV-KS was in the mouth; in 6 patients the initial presentation of HIV-KS was concurrently in the mouth and on the skin; 10 patients (27%) developed cutaneous HIV-KS before the appearance of oral HIV-KS (Table 1), on average 4.5 weeks before the diagnosis of their oral HIV-KS. Three patients developed cutaneous HIV-KS after their diagnosis of oral HIV-KS on average 4.3 weeks later, and 18 patients (49%) did not develop cutaneous HIV-KS. 

The CD4+ T-cell counts were obtained only for 33 patients, the mean CD4+ T-cell count being 107 cells/mm^3^ (Table 1). At the time of diagnosis of oral HIV-KS, twelve patients (32%) had concomitant oral candidiasis, one had concomitant oral hairy leukoplakia, and one had concomitant necrotizing gingivitis (Table 1). Nineteen patients (51%) had concurrent infection with *Mycobacterium tuberculosis* (TB), one had gonorrhoea, and one had bronchitis. 

At the time of oral HIV-KS diagnosis, eight of the 37 patients had solitary oral lesions and 29 (78%) had multiple lesions affecting one or more oral sites (Table 1). There were significantly more patients with multiple oral HIV-KS lesions than patients with single oral HIV-KS lesions (*P* = 0.0003). All 37 patients collectively had 93 separate oral HIV-KS lesions. The clinical appearance and the size of the lesions are documented in Table 1. 

Twenty-eight oral HIV-KS lesions (30%) affected the gingiva, 24 (26%) affected the hard palate, 22 (24%) affected the oropharynx (upper and lower retromolar areas, and the soft palate), 14 (15%) affected the alveolar mucosa, and five affected the dorsum of the tongue (Table 2). The oral lesions ranged in colour from pink to red and from bluish-purple to deep brown. 

When the clinical features of oral HIV-KS and CD4+ T-cell counts of patients with HIV-KS at the time of oral HIV-KS diagnosis (Table 1) were compared between males and females using the Chi-squared* test*, there were no statistically significant differences identified, except for the size of the lesions. The percentage of lesions <10 mm was significantly lower in females than in males (*P* = 0.007), whereas the percentage of lesions ≥10 mm ≤50 mm was significantly higher in females than in males (*P* = 0.004). 

### 3.1. HIV Infection and HAART

The CD4+ T-cell counts were available for only 33 of the 37 patients (Table 3). In these patients the average CD4+ T-cell count at the time of oral HIV-KS diagnosis was 107 cells/mm^3^. Seventeen patients (46%) were concurrently diagnosed with HIV infection and oral KS and CD4+ T-cell counts were available for only 14 of these 17 patients. The mean CD4+ T-cell counts of these patients (130 cells/mm^3^) was not statistically different (*P* = 0.296) from the 19 patients who were diagnosed with HIV infection before developing oral HIV-KS (90 cells/mm^3^) (Table 3). The difference between the average CD4+ T-cell count at the time of HIV diagnosis (164 cells/mm^3^) and at the time of oral HIV-KS diagnosis (90 cells/mm^3^) was not statistically significant (*P* = 0.11). 

Thirty patients (81%) were HAART-naïve, and seven patients had already been receiving HAART at the time of oral HIV-KS diagnosis. CD4+ T-cell counts were available for 26 of the 30 HAART-naïve patients at the time of oral HIV-KS diagnosis. Their average CD4+ T-cell count of 114 cells/mm^3^ was not statistically different (*P* = 0.606) from the CD4+ T-cell counts of the seven patients who were on HAART at the time of oral HIV-KS diagnosis (90 cells/mm^3^). Ten patients (27%) started HAART around the time of or soon after their oral HIV-KS diagnosis. 

### 3.2. Facial Lymphoedema

Nine patients, five males, and four females had facial lymphoedema. Three patients presented with facial lymphoedema at the time their oral HIV-KS were diagnosed, and six patients subsequently developed facial lymphoedema on average 2.3 weeks after the diagnosis of oral HIV-KS. All patients with facial lymphoedema had multifocal exophytic oral HIV-KS lesions and their average CD4+ T-cell count at the time of oral HIV-KS diagnosis was 28 cells/mm^3^ (CD4+ T-cell counts were available for eight of the nine patients) compared to 133 cells/mm^3^ for those patients without such facial lymphoedema (Table 3). This difference in the average CD4+ T-cell counts was statistically significant (*P* = 0.01). All nine patients with facial lymphoedema died very soon after their oral HIV-KS occurred, on average within two weeks, regardless whether they were receiving HAART. No significant difference was observed between the average CD4+ T-cell count of females (31 cells/mm^3^) and males (24 cells/mm^3^) with facial lymphoedema (*P* = 0.54). 

### 3.3. Immune Reconstitution Inflammatory Syndrome (IRIS)-Associated Oral HIV-KS

One patient had IRIS-associated HIV-KS. The CD4+ T-cell count of this female patient at the time of her HIV diagnosis was 9 cells/mm^3^, and she developed IRIS-associated HIV-KS, four weeks after she started HAART. 

### 3.4. The Course of Oral HIV-KS

Nine patients were lost to follow-up. Of the remaining 28 patients, oral HIV-KS lesions increased in number and/or in size in 21 patients (75%), remained stable or shrunk in four patients, and resolved in three patients.

Twenty-one patients died during the observation period, on average 13.6 weeks from the time of oral HIV-KS diagnosis (Table 4). Eleven of these 21 patients (52%) did not receive HAART nor any other treatment for their oral HIV-KS. The average time from oral HIV-KS diagnosis to their death was 20 weeks. Eight of the 21 patients who died were on HAART as a sole modality of treatment, and they died on average of 4.4 weeks after their HIV-KS diagnosis. Two patients were treated with HAART in combination with local cytotoxic chemotherapy and they died on average 20.5 weeks after their HIV-KS diagnosis. 

Of those seven patients who survived, in three there was resolution of the oral HIV-KS (one had IRIS-associated HIV-KS and was treated with HAART in combination with systemic cytotoxic chemotherapy and surgery; one was treated with HAART and systemic cytotoxic chemotherapy; one with HAART and surgery). In the remaining four patients, the oral HIV-KS lesions remained unchanged or shrunk. These patients were treated with HAART or with HAART in combination with local cytotoxic chemotherapy. 

The average CD4+ T-cell count of the patients who were alive at the end of the study observation period was 166 cells/mm^3^ at oral HIV-KS diagnosis (Table 4), while the average CD4+ T-cell count of the patients who died during the observation period was 64 cells/mm^3^ at the time of oral HIV-KS diagnosis. Statistically, the difference in the CD4+ T-cell count between these two groups of patients was significant (*P* = 0.016). 

## 4. Discussion

**Figure 1:**
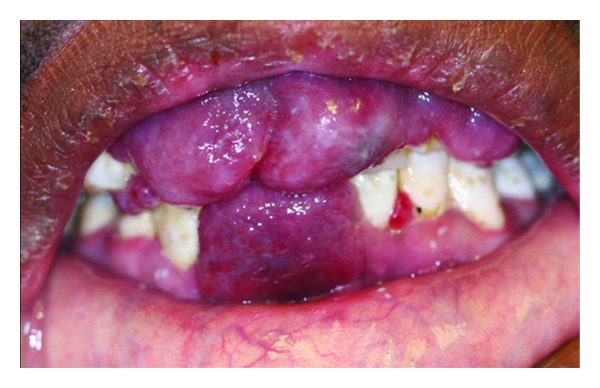
Exophytic oral HIV-KS lesions on the anterior maxillary and mandibular buccal gingiva of a 31-year-old male with a CD4+ T-cell count of 5 cells/mm^3^.

**Figure 2:**
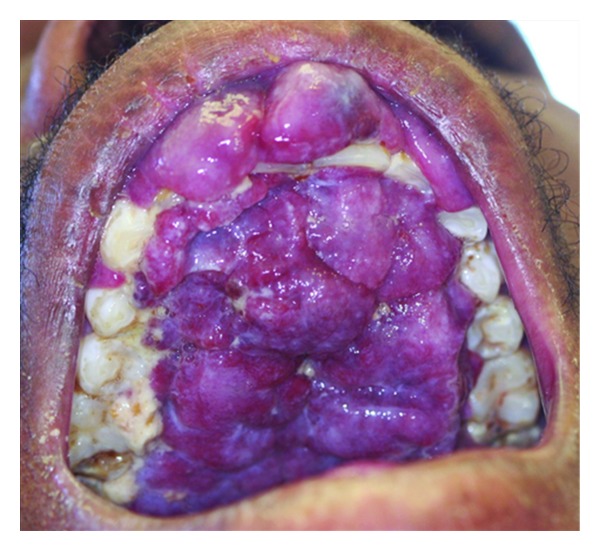
Exophytic confluent oral HIV-KS lesion on the hard palate in a 31-year-old male patient with a CD4+ T-cell count of 5 cells/mm^3^.

**Figure 3:**
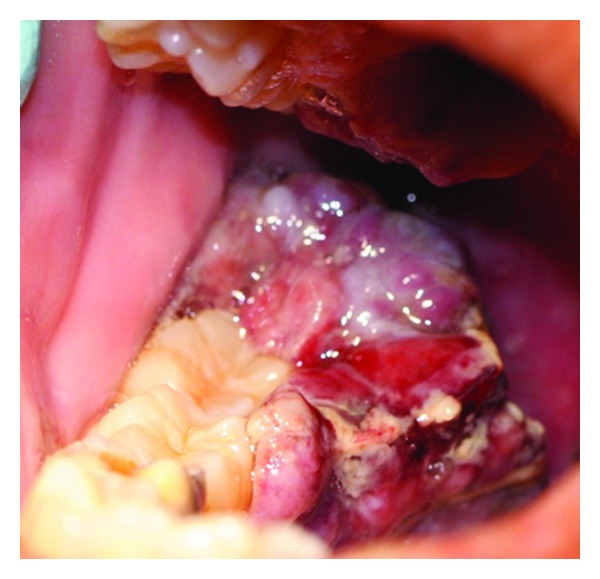
Exophytic oral HIV-KS lesion on the lower right retromolar area extending into the oropharynx in a 29-year-old female patient with a CD4+ T-cell count of 49 cells/mm^3^. The patient died six weeks after her oral HIV-KS diagnosis.

**Figure 4:**
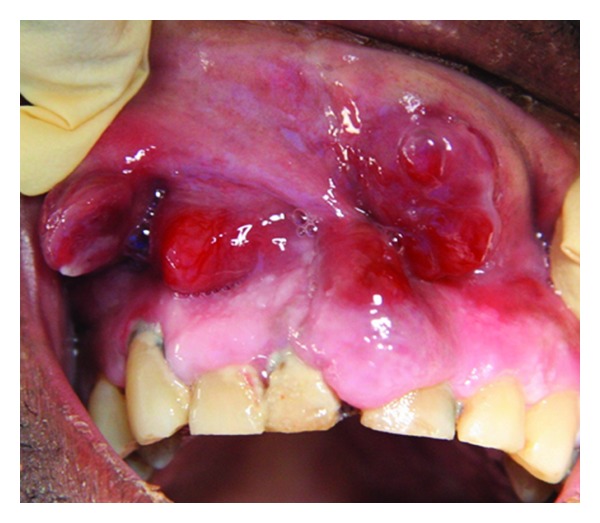
Exophytic oral HIV-KS lesions on the alveolar and labial mucosa in a 54-year-old male with a CD4+ T cell count of 258 cells/mm^3^. The patient died 15 weeks after his oral HIV-KS diagnosis.

**Figure 5:**
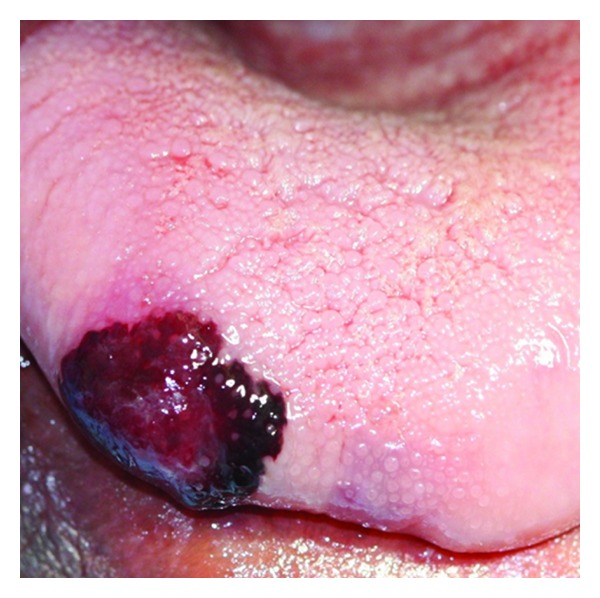
Macular/nodular lesion on the dorsum of the tongue in a 44-year-old female patient with a CD4+ T-cell count of 13 cells/mm^3^. The patient died five weeks after the diagnosis of her oral HIV-KS.

There was a significantly higher number of patients with multiple oral lesions at the time of oral HIV-KS diagnosis than patients who had single lesions (Table 1). With decreasing order of frequency, the gingiva, hard palate, oropharynx (upper and lower retromolar area, and soft palate), alveolar mucosa, and the dorsum of the tongue (Figures 1, 2, 3, 4, and 5) were the sites most commonly affected (Table 2), conforming to other reports in the literature [4, 21]. In none of the 37 patients included in this study was the floor of the mouth or the ventral/lateral surface of the tongue affected. It is unknown why HIV-KS has the tendency to affect only certain oral sites, but not others.

Forty six percent (46%) of the patients with oral HIV-KS in this study cohort did not know their HIV-serostatus at the time of oral HIV-KS diagnosis, implying that oral KS in the Ga-Rankuwa area in South Africa may serve as an indicator of HIV infection. Although the prevalence of HHV8 infection in South Africa is relatively high [22, 23], there were no recorded cases of oral KS in HIV-seronegative subjects during the study period, suggesting that endemic African KS is not frequent in the Ga-Rankuwa area of South Africa.

Seven patients were on HAART at the time of oral HIV-KS diagnosis and although at this time their average CD4+ T-cell count (90 cells/mm^3^) was lower than the average CD4+ T-cell count of the HAART-naïve patients (114 cells/mm^3^) (Table 3), this difference was not statistically significant. This seemingly surprising finding that the patients on HAART had a lower CD4+ T-cell count than the HAART-naïve patients could be attributed to the fact that in South Africa, HIV-seropositive persons who rely on provincial (governmental) services for their medical care have to abide by an official policy that HAART can be provided only when their CD4+ T-cell count has dropped below 200 cells/mm^3^, and that some people who have medical conditions suggestive of HIV disease are reluctant to undergo serological testing for HIV and often prefer to be treated by traditional healers. As a result, HIV infection is often diagnosed and HAART is often introduced only late in the course of their HIV disease when the CD4+ T-cell counts have already fallen substantially below 200 cells/mm^3^.

As a consequence of HAART being introduced only when the CD4+ T-cell count is already very low, there will be a lower level of reconstitution of the CD4+ T-cell number compared to the level of reconstitution when HAART is started at a higher CD4+ T-cell count [8, 24–28]. In addition, in provincial (governmental) medical facilities in South Africa, as in other countries in sub-Saharan Africa, monitoring the effectiveness of HAART is not always as efficient as it is in developed countries due to limited resources [28, 29]. All these factors may explain why the average CD4+ T-cell count of our study cohort was low at the time of oral HIV-KS diagnosis, why the number of HAART-naïve patients was high, and why the CD4+ T-cell counts of patients on HAART was also low. 

It has been reported that in 22% of HIV-seropositive subjects with KS, the initial presentation of HIV-KS is in the mouth, and that in up to 70% of subjects with HIV-KS, the mouth will sooner or later be affected [12, 13]. As our study was designed to include only patients with oral HIV-KS regardless of whether they had extraoral KS, our findings cannot be compared to the findings of other studies reported in the literature in which the inclusion criterion was that of patients having HIV-KS, but who may or may not have had oral HIV-KS. However, our data show that in 21 patients (57%) the initial presentation of HIV-KS was in the mouth, and that in six patients the initial presentation of HIV-KS occurred concurrently in the mouth and on the skin. Ten patients (27%) developed cutaneous KS before and three after oral HIV-KS diagnosis. Eighteen patients (49%) with oral HIV-KS did not develop cutaneous HIV-KS during the observation period. These findings emphasise that in many cases the mouth may be the only body site affected by HIV-KS. 

Facial lymphoedema may occur before, at the time of, or after oral HIV-KS is diagnosed. Facial lymphoedema which develops in parallel with the progression of oral HIV-KS disease is an indicator of poor prognosis [7, 8] and foretokens death [30]. In this study, the nine patients who had facial lymphedema had extensive exophytic oral lesions, severe immunosuppression, and average CD4+ T-cell count of 28 cells/mm^3^ and all died very soon after the onset of facial lymphedema, regardless of HAART. 

The pathogenic mechanisms that cause facial lymphoedema in association with oral HIV-KS are obscure. However, as oral KS lesions and oral fluids of HIV-seropositive subjects carry a high HHV8 load, and as advanced exophytic oral HIV-KS lesions have a higher HHV8 load than initial maculopapular lesions [31], it is possible that in the presence of exophytic oral lesions, lymphatic obstruction secondary to HHV8-induced proliferation of endothelial cells, and/or compression of lymphatics by rapidly progressing oral HIV-KS, will bring about leakage of protein-rich fluid into the interstitial spaces, promoting the development of facial lymphoedema [32, 33]. Therefore, it is likely that treating exophytic oral HIV-KS lesions with cytotoxic chemotherapy may result in the shrinkage of oral lesions with the subsequent decrease in HHV8 load in the affected tissues, thus reducing the risk of developing facial lymphoedema. This possibility needs further investigation.

To the best of our knowledge, this is the first report in the literature documenting the prevalence of IRIS-associated oral HIV-KS in a population of patients with oral HIV-KS regardless of whether they had extraoral KS. One patient in this case series had IRIS-associated HIV-KS. This patient was successfully treated with systemic cytotoxic chemotherapy and surgical excision. A comprehensive description of the case report of this patient has been published previously [34]. At the time of writing this paper, the patient was still alive 5.5 years after the treatment of IRIS-associated oral HIV-KS and currently her CD4+ T-cell count is 383 cells/mm^3^. This is in line with reports in the literature documenting that IRIS-associated oral HIV-KS responds well to conventional therapy [35, 36]. 

During the study observation period, 21 of the 37 patients (57%) died, on average within 13.6 weeks from the time of oral HIV-KS diagnosis; nine were lost to follow-up; 7 patients survived (average period of follow-up of 91 weeks). The average CD4+ T-cell counts of the patients who were alive at the end of the study observation period was 166 cells/mm^3^ at oral HIV-KS diagnosis and the CD4+ T-cell counts of the patient who died was 64 cells/mm^3^, at oral HIV-KS diagnosis. The difference in these CD4+ T-cell counts was statistically significant (*P* = 0.016). This suggests that a low CD4+ T-cell count at the time of oral HIV-KS diagnosis is a strong indicator of poor prognosis. Unfortunately, we were unable to determine the cause of death of our patients and therefore it is unknown whether they died as a direct consequence of their HIV-KS disease. 

Of those who survived, in three patients oral HIV-KS completely resolved following various treatment modalities. One of these patients was treated with HAART in combination with systemic cytotoxic chemotherapy and surgery, one with HAART in combination with cytotoxic chemotherapy, and one patient with HAART and surgery. In four patients, the oral HIV-KS remained unchanged or shrunk. These patients were treated with HAART or with HAART in combination with local cytotoxic chemotherapy.

HAART is used to treat HIV infection. Even though effective HAART does not directly influence HHV8 replication, by reducing HIV load with subsequent improvement in the host-immune status, it indirectly brings about a decrease in the incidence and prevalence of HIV-KS and an improvement in the clinical manifestation of pre-existing HIV-KS disease [29]. HAART may also have a direct anti-angiogenic effect on KS. However, HAART does not ensure that HIV-KS will not develop, and despite HAART, KS remains the most frequent HIV-associated neoplasm [30].

In this study, seven patients have been on HAART at least four weeks before their oral HIV-KS diagnosis, confirming that HIV-seropositive subjects on HAART may develop KS. Eight of the 21 patients who died during the observation period were on HAART as a sole modality of treatment. These patients died on average 4.4 weeks after their oral HIV-KS diagnosis. During this period, they experienced worsening of their oral HIV-KS disease. This suggests that although introduction of HAART should be the first line of therapy for HAART-naïve HIV-seropositive subjects with oral KS, HAART by itself may not be effective in controlling oral HIV-KS disease. Two of the 21 patients who died were concurrently treated with HAART and with local cytotoxic chemotherapy.

Eleven of the 21 patients (52%) who died during the observation period received neither HAART nor any other treatment for their oral HIV-KS. The average time from oral HIV-KS diagnosis to their death was 20 weeks. The paradoxical finding that in this study HAART-naïve patients lived longer than patients on HAART might be attributed to skewed statistics associated with the small number of patients, or to ineffective HAART that was started late in the course of oral HIV-KS disease when the CD4+ T-cell count had already fallen very low. 

The small number of patients who received treatment for HIV-KS in this study prevents drawing conclusions regarding what is the best treatment approach to control the progression of oral HIV-KS and to improve the prognosis of the patients. However, as reported elsewhere [37], it seems that exophytic oral HIV-KS lesions are best treated with HAART and systemic cytotoxic chemotherapy, and once the lesions have shrunk and become surgically accessible, they should be excised.

In developed countries, HIV-KS predominantly affects males. However, in many resource poor countries in sub-Saharan Africa where HIV infection is endemic and young females aged 15–24 years are more frequently infected with HIV than males, there is almost an identical incidence of HIV-KS in males and females [19, 38], and at the time of HIV-KS diagnosis females present with a more advanced disease than males [19, 39]. In our study, more females than males were affected by oral HIV-KS and although not statistically significant, females had a lower average CD4+ T-cell count (85 cells/mm^3^) than males (141 cells/mm^3^) at the time of oral HIV-KS diagnosis, in line with other studies documenting that females with HIV-KS have more severe immunodeficiency than males with HIV-KS [39]. However, in contrast to other studies reporting that females are younger than males at the time of HIV-KS diagnosis [39, 40], the age of the females (33 years) and males (34 years) in our cohort was very similar. 

In spite the fact that the CD4+ T-cell counts of females were lower than males at oral HIV-KS diagnosis, the differences between the percentage of males and females who survived or died were not statistically significant. Other reports in the literature also note that gender differences do not influence survival of patients with HIV-KS [19].

Two patients were children. Both were boys, aged 10 and 11 years. This supports data reported from other areas of sub-Saharan Africa, that children are not uncommonly affected by HIV-KS. In fact, in Zimbabwe, the most frequent malignancy in children between 1 and 14 years of age is HIV-KS [38]. This is probably owing to the high prevalence of HHV8 and HIV coinfection in African children in this part of the world. HIV-seropositive children with advanced oral KS have a particularly aggressive course of disease with a poor prognosis [41]. In sub-Saharan Africa, oral involvement in HIV-seropositive children with KS is common.

Thirty-eight percent (38%) of the patients in this study had common HIV-associated oral diseases which presented concurrently with oral HIV-KS (Table 1). It is probable that these diseases were associated with the low CD4+ T-cell counts of the patients, and not with the oral HIV-KS. However, one cannot exclude the possibility that oral HIV-KS-associated cytokine dysregulation may favour the development of other HIV-associated oral lesions and that some HIV-associated oral diseases may further dysregulate the cytokine milieu in the affected oral tissues, thus promoting KS tumourigenesis [42, 43]. 

## 5. Summary

In the Ga-Rankuwa area of South Africa where HIV-KS is prevalent, oral KS affects similarly males and females. In this population, a low CD4+ T-cell count at the time of oral HIV-KS diagnosis is associated with a poor prognosis. The development of facial lymphoedema during the course of HIV-KS disease portends a very poor prognosis. Owing to the small number of patients who received treatment, it was not possible to determine what the best treatment modality was for oral HIV-KS. 

## Figures and Tables

**Table 1:** Clinical and laboratory features of the patients at the time of oral HIV-KS diagnosis.

	Males	Females	Total
Number of patients (%)	**17 (46%)**	**20 (54%)**	**37 (100%)**
Age (years)			
Mean	**34**	**33**	**33.4**
*Range *	*11–55*	*19–46*	*11–55*
*Standard deviation *	*12.53*	*7.69*	*10.08*
Tobacco usage (%)	**7 (41%)**	**2 (10%)**	**9 (24%)**
Number of patients in whom the initial presentation of HIV-KS was in the mouth	**8 (47%)**	**13 (65%)**	**21 (57%)**
Number of patients in whom the initial presentation of HIV-KS was concurrently in the mouth and skin	**3 (18%)**	**3 (15%)**	**6 (16%)**
Number of patients who developed cutaneous HIV-KS before oral HIV-KS diagnosis	**6 (35%)**	**4 (20%)**	**10 (27%)**
Other oral lesions present			
Pseudomembranous candidiasis	**7 (41%)**	**5 (25%)**	**12 (34%)**
Hairy leukoplakia		**1 (5%)**	**1 (2%)**
Necrotizing gingivitis	**1 (6%)**		**1 (2%)**

Total number of patients	**8 (47%)**	**6 (30%)**	**14 (38%)**

Average CD4+ T-cell count at KS diagnosis (data available for 33 patients) [cells/mm^3^]	**141**	**85**	**107**
* Range*	*12–409 *	*13–261*	*12–409*
* Standard deviation*	*117.40*	*77.99*	*106.99*
Number of patients diagnosed with HIV infection and oral KS at the same time	**10 (59%)**	**7 (35%)**	**17 (46%)**
Number of patients who were diagnosed with HIV infection before the diagnosis of oral KS	**7 (41%)**	**13 (65%)**	**20 (54%)**
Number of patients with single oral HIV-KS lesions	**5 (29%)**	**3 (15%)**	**8 (22%)**
Number of patients with multiple oral HIV-KS lesions	**12 (71%)**	**17 (85%)**	**29 (78%)**
Lesion phenotype			
Number of macular lesions	** 9 (20%)**	**8 (17%)**	**17 (18%)**
Number of papular lesions	**10 (22%)**	**11 (23%)**	**21 (23%)**
Number of nodular lesions	**16 (36%)**	**17 (35%)**	**33 (35%)**
Number of exophytic lesions	**10 (22%)**	**12 (25%)**	**22 (24%)**

Total number of lesions	**45 (100%)**	**48 (100%)**	**93 (100%)**

Lesion size			
Number of lesions <10 mm	**15 (33%)**	**5 (10%)**	**20 (22%)**
Number of lesions ≥10 mm ≤50 mm	**25 (56%)**	**40 (83%)**	**65 (70%)**
Number of lesions >50 mm	**5 (11%)**	**3 (7%)**	**8 (8%)**

Total number of lesions	**45 (100%)**	**48 (100%)**	**93 (100%)**

**Table 2:** Oral sites affected by oral HIV-KS in relation to gender.

	Males	Females	Total (%)
Gingiva	**13 (29%)**	**15 (31%)**	**28 (30%)**
Upper gingiva	7 (16%)	10 (21%)	17 (18%)
Lower gingiva	6 (13%)	5 (10%)	11 (10.8%)
Hard palate	**11 (24%)**	**13 (27%)**	**24 (26%)**
Oropharynx	**10 (22%)**	**12 (25%)**	**22 (24%)**
Alveolar mucosa	**8 (18%)**	**6 (13%)**	**14 (15%)**
Upper alveolar mucosa	4 (9%)	3 (6%)	7 (7.55%)
Lower alveolar mucosa	4 (9%)	3 (6%)	7 (7.55%)
Dorsum of tongue	**4 (9%)**	**1 (2%)**	**5 (5%)**

Total number of lesions	**45 (100%)**	**48 (100%)**	**93 (100%)**

**Table 3:** CD4+ T-cell counts (cells/mm^3^) of the participants.

CD4+ T-cell counts of the patients	Males	Females	Average
At the time of oral HIV-KS diagnosis (33 patients)	**141 (14)**	**85 (19)**	**107 (33)**
* Standard deviation*	*117.40*	*77.99*	*106.99*
Who were simultaneously diagnosed with HIV and oral KS (14 patients)	**163 (7)**	**97 (7)**	**130 (14)**
* Standard deviation*	*155.64*	*85.95*	*125.63*
Who were diagnosed with HIV infection before developing oral HIV-KS, at the time of HIV diagnosis (14 patients)	**210 (6)**	**129 (8)**	**164 (14)**
* Standard deviation*	*167.27*	*160.21*	*162.14*
Who were diagnosed with HIV infection before developing oral HIV-KS, at the time of oral HIV-KS diagnosis (19 patients)	**119 (7)**	**74 (12)**	**90 (19)**
* Standard deviation*	*112.92*	*75.55*	*90.75*
Receiving for some time HAART, at HIV-KS diagnosis (7 patients)	**160 (1)**	**78 (6)**	**90 (7)**
* Standard deviation*	*0*	*71.49*	*72.19*
Who were HAART-naïve at oral HIV-KS diagnosis (26 patients)	**140 (13)**	**87 (13)**	**114 (26)**
* Standard deviation*	*137.98*	*83.75*	*114.97*
Who had facial lymphoedema during their course of oral HIV-KS (8 patients)	**24 (4)**	**31 (4)**	**28 (8)**
* Standard deviation*	*14.66*	*15.75*	*14.61*
Who did not have lymphoedema during the course of oral HIV-KS (25 patients)	**188 (10)**	**96 (15)**	**133 (25)**
* Standard deviation*	*129.50*	*82.60*	*111.33*

**Table 4:** Mortality and survival in relation to oral HIV-KS.

	Males	Females	Total
Mortality			
Number of patients who died	11 (85%)	10 (66%)	21 (75%)
Average time of death from oral HIV-KS diagnosis	15 weeks	12.1 weeks	13.6 weeks
Average CD4+ T cell count (cells/mm^3^) at oral HIV-KS diagnosis	75	54	64
Survival			
Number of patients who survived	2 (15%)	5 (33%)	7 (25%)
Average follow-up time	76 weeks	106 weeks	91 weeks
Average CD4+ T cell count (cells/mm^3^) at oral HIV-KS diagnosis	258	129	166
